# Effectiveness and safety of caplacizumab in acquired thrombotic thrombocytopenic purpura: health technology assessment and classification according to the methodology established in Colombia

**DOI:** 10.1017/S0266462323000442

**Published:** 2023-07-21

**Authors:** Jahir A. Soto-Mora, Lina M. Gómez-Espitia, Pieralessandro Lasalvia, Camilo A. Castellanos Moreno, Carol A. Casallas Vanegas, Sergio A. Londoño Gutiérrez

**Affiliations:** 1 Department of Evidence-Based Medicine, NeuroEconomix, Bogotá, Colombia; 2 Department of Health Economics, NeuroEconomix, Bogotá, Colombia; 3 Sanofi, Bogotá, Colombia

**Keywords:** health technology assessment, caplacizumab, thrombotic thrombocytopenic purpura

## Abstract

**Objectives:**

Acquired thrombotic thrombocytopenic purpura (aTTP) is a rare hematological disease whose clinical management includes caplacizumab along with plasma exchange and immunosuppression, according to international guidelines. Caplacizumab has been available in Colombia since 2022. This study seeks to determine the therapeutic classification of caplacizumab according to the methodology of the Instituto de Evaluación Tecnológica en Salud.

**Methods:**

The classification was carried out through a deliberative process following the modified Delphi technique, with a panel of experts, made up of four hemato-oncologists, a pharmaceutical chemist, and a patient. The results of effectiveness and safety obtained through a systematic review, therapeutic thresholds (clinical significance), and degree of acceptability (willingness to use the technology) were used for the classification.

**Results:**

Fourteen effectiveness and safety outcomes were submitted for the classification process. Caplacizumab showed clinical significance for some effectiveness outcomes, was not considered inferior in terms of safety, and displayed acceptability of use. Through consensus, the panel determined that caplacizumab plus the standard regimen is superior to the standard regimen in terms of treatment response and composite outcome, and no different for the other effectiveness and safety outcomes. Likewise, in overall terms, the panel determined that caplacizumab together with the standard regimen is superior to the standard regimen.

**Conclusion:**

Treatment with caplacizumab together with the standard regimen was considered superior to the standard regimen for the treatment of patients with aTTP, as it showed clinically significant benefits in critical outcomes for decision making, and a safety profile no different to its comparator.

## Introduction

Thrombotic thrombocytopenic purpura (TTP) is a well-defined entity which consists of a heterogeneous group of thrombotic microangiopathies (1). This is considered a rare hematologic disease, characterized by the presence of microangiopathic hemolytic anemia, thrombocytopenia, and systemic vascular events (2).

TTP is understood to be a state of severe ADAMTS13 protease deficiency, caused by both genetic abnormalities and autoantibodies that affect the function of ADAMTS13 (1). TTP is divided into two main types, based on the mechanism of ADAMTS13 deficiency: congenital (inherited) and immune-mediated (acquired) (1–4). Acquired thrombotic thrombocytopenic purpura (aTTP) is the most common type, representing between 90-95 percent of cases (5–8). This type of TTP has a known annual incidence of three to eleven cases per million people (9). As for the prevalence, it is difficult to estimate given the nature of the disease. However, countries with robust patient registries, such as the United States, France, and Spain, have estimated a prevalence of 19, 13, and 21 patients per million, respectively (10–12).

An acute episode of aTTP is a medical emergency that requires urgent diagnosis and treatment (1;4;6;13). Untreated, mortality can reach up to 90 percent (3), and delays in treatment can lead to significant morbidity and mortality (1;4;6;14;15). Historically, the initial standard treatment consisted of daily plasma exchange and immunosuppression with glucocorticoids and/or rituximab in some cases (13;16;17), in order to provide adequate levels of ADAMTS13 and suppress the production of anti-ADAMTS13 autoantibodies (3;6). Recently, the clinical guidelines have included caplacizumab together with plasma exchange and immunosuppression as the first-line treatment for patients with aTTP (7;18;19).

Caplacizumab is the first drug specifically approved to treat aTTP by the principal regulatory bodies at a worldwide level, such as the FDA and the EMA (20;21). This molecule is a humanized bivalent nanoantibody that inhibits the interaction between the von Willebrand factor and platelets, preventing platelet adhesion mediated by high-molecular-weight von Willebrand factor multimers. With this mechanism of action, caplacizumab offers a new focus of treatment, preventing the development of potentially fatal microvascular thrombosis that can occur in the process of the disease (22).

Caplacizumab is approved for use and commercialization in Colombia since January 2022. The introduction of a new alternative requires knowledge of the comparative benefits, harm, or usefulness of treatment of caplacizumab together with the standard regimen, versus the standard regimen, through a health technology assessment (HTA) to determine the value and additional benefit of the new therapy, providing information to guide decision making in health care and the use of health system resources.

In Colombia, the Institute for Health Technology Assessment (Instituto de Evaluación Tecnológica en Salud, IETS) is the entity in charge of establishing the methodological and technical requirements for the development of HTAs for regulatory purposes (23). Recently, the IETS updated the manuals that guide the elaboration of HTAs (23–25). In these manuals, an additional requirement for the assessments is the classification of the health technology. This new process aims to determine whether the technology of interest is superior, no different, or inferior to its comparator or comparators, based on the results of the effectiveness and safety evaluation, therapeutic value thresholds, and degree of acceptability of the effect of the technology. The present study aimed to evaluate the effectiveness and safety of caplacizumab in conjunction with the standard regimen compared with the standard regimen in the treatment of patients with aTTP, and to classify the technology of interest according to the methodology established in Colombia.

## Methods

This review was carried out following the methodological and technical guidelines established in the IETS manuals of Colombia (23–25), and the recommendations of the PRISMA statement and the Cochrane manual (26;27).

In general, the steps followed to perform the evaluation and classification of the technology of interest were as follows: (i) definition of the research question, (ii) definition of the eligibility criteria, (iii) screening and selection of studies, (iv) risk of bias assessment, (v) data extraction, (vi) evidence synthesis, (vii) assessment of the quality or body of evidence for each outcome (GRADE), (viii) determination of the threshold of therapeutic value, and (ix) classification of the technology.

### Proposal of Research Question

The research question in PICOT format was refined through consultation with two hematology specialists, a patient with the condition of interest, and four methodology experts in the evaluation and synthesis of scientific evidence (Table 1) (23).

**Table 1.** Research question

Population	Adults (greater than or equal to 18 years) Diagnosis of aTTP In the case of females, that they were not pregnant
Intervention	Caplacizumab + plasma exchange + glucocorticoids, with or without another immunosuppressant (standard regimen)
Comparison	Plasma exchange + glucocorticoids, with or without another immunosuppressant (standard regimen)
Outcomes	Effectiveness/efficacy Response time to treatment Response to treatment Clinical remission Recurrence Relapse Exacerbation Refractoriness Quality of life Mortality Number of days of plasma exchange Volume of plasma exchange Number of days of hospitalization Number of days in intensive care unit Safety Any adverse event Serious adverse events Serious adverse events related to the drug Any adverse event related to the drug Discontinuation or interruption of treatment due to an adverse event Specific adverse events of interest (bleeding)
Type of study	Randomized clinical trials Real-world observational studies

aTTP, acquired thrombotic thrombocytopenic purpura.

The relative importance of the outcomes was rated by the group of clinical experts and the patient, using a nine-point Likert scale, following the methodology proposed by the GRADE group (22). Those outcomes whose mean score was equal to or greater than four (important and critical) were considered, with all the outcomes of interest being classified as critical (Supplementary Table 1).

### Data Sources, Selection of Studies, and Data Extraction

A systematic search was performed in the databases Medline, Embase, the Cochrane Central Register of Controlled Trials, and WHO International Clinical Trials. The search was performed for all databases from their inception date to 25 February 2022. The search strategies used for each database are presented in Supplementary Table 2. In addition, a manual search was performed reviewing the bibliography of the selected studies and identifying additional references using the “Similar articles” tool of PubMed and “Matrix of Evidence” by Epistemonikos.

Studies that met the following inclusion criteria were considered: (i) randomized clinical trials (RCTs) and real-world observational studies which evaluated the intervention of interest; (ii) studies that included adult patients (greater than or equal to 18 years) with a diagnosis of aTTP; (iii) studies that reported at least one result of interest; and (iv) studies published in Spanish or English. Studies published solely in the format of an abstract or a letter to the editor were excluded.

Screening was performed independently by two reviewers (J.A.S.-M. and L.M.G.-E.) based on title and abstract; the selected references were retrieved and reviewed in full text by the same reviewers to define their inclusion, resolving disagreements by consensus.

Data extraction was performed in a standardized format, recording the primary author, year, treatments, country, age, population, sample size, and results of interest. The process was carried out by one reviewer, with quality control by a second reviewer, comparing the results included in the evaluation report with those presented in the original publications.

The risk of bias assessment was performed by one reviewer and checked by a second reviewer. The Cochrane RoB2 risk of bias tool was used for RCTs (27) and the JBI Critical Appraisal Checklist for Cohort Studies tool (28) for observational studies. The certainty of the evidence for each outcome was assessed using the GRADE methodology (29).

### Therapeutic Value Threshold, Acceptability, and Classification of the Technology

HTAs in Colombia require the classification of the technology (24). The classification process is based on efficacy and safety results, therapeutic thresholds, and the degree of acceptability of the effect of the technology.

The therapeutic threshold is the clinical benefit that the technology will provide versus its comparator beyond the statistical significance obtained and considering other elements in context (24). The determination of the threshold for each outcome was performed through a deliberative process using a modified Delphi (25). The panel of experts consisted of four medical specialists in hematology, two of them also specialists in epidemiology, a professional in pharmaceutical chemistry, and a patient with the condition of interest.

To define the thresholds, the following steps were performed (23):

1. The panel was informed of the number of patients with the outcome in the comparator treatment group (standard regimen) identified in the literature, using GRADE tables and pictograms.
2. They were asked for the minimum number of patients (increase/reduction) with the event/outcome in the intervention group of interest (caplacizumab), to determine if the effect is clinically significant. This number (the threshold) was obtained in a discussion with the participants through different guiding questions.
3. The threshold value obtained was voted on to determine the group’s level of agreement. The panel voted individually and anonymously through an *online* form, using a nine-point Likert scale, where one is “Totally disagree” and nine is “Totally agree.”
4. The results were analyzed in terms of percentage and median with a 95 percent confidence interval (95 percent CI). The threshold was approved when more than 80 percent of the participants voted between seven and nine and/or a median of eight was obtained with a 95 percent CI between seven and nine.
5. The results identified in the literature on the technology of interest were presented using GRADE tables and pictograms to analyze whether they exceeded the threshold established by consensus and determine clinical significance. To exceed the threshold, the point estimate and lower CI had to be equal to or greater than the threshold.

With the definition of thresholds for each of the outcomes and the clinical significance, the acceptability of the effect of the technology was then established (24). Each of the experts was asked to declare their willingness or not to use the technology of interest, through an *online* form that included a summary of the scientific evidence presented and a synthesis of the results of the therapeutic threshold. If 80 percent or more of the participants reported willingness to use the technology, the acceptability of the effect of the technology was determined.

Once the clinical significance and the degree of acceptability were established, a new deliberative process was carried out to determine the classification of the technology. For this process, the following steps were performed (23;24).

1. The panel was presented with the input matrix for technology classification, which contained the relative and absolute estimator of the technology effect (calculated with GRADEpro) with their respective 95 percent CI, the certainty of evidence, the therapeutic threshold, the estimator with respect to the therapeutic threshold, and the acceptability of the effect for each of the outcomes (Supplementary Table 3).
2. The panel was asked to determine for each result if the technology of interest was superior or not to its comparator, according to the input matrix for the classification and the categories established by the IETS (Supplementary Table 4).
3. The established category was put to a vote to determine consensus. The panel voted individually and anonymously through an *online* form, using a nine-point Likert scale, where one is “Totally disagree” and nine is “Totally agree.”
4. The results were analyzed in terms of percentage and median with a 95 percent confidence interval (95 percent CI). The classification category was approved when more than 80 percent of the participants voted between seven and nine and/or a median of eight was obtained with a 95 percent CI between seven and nine.

## Results

**Figure 1.**
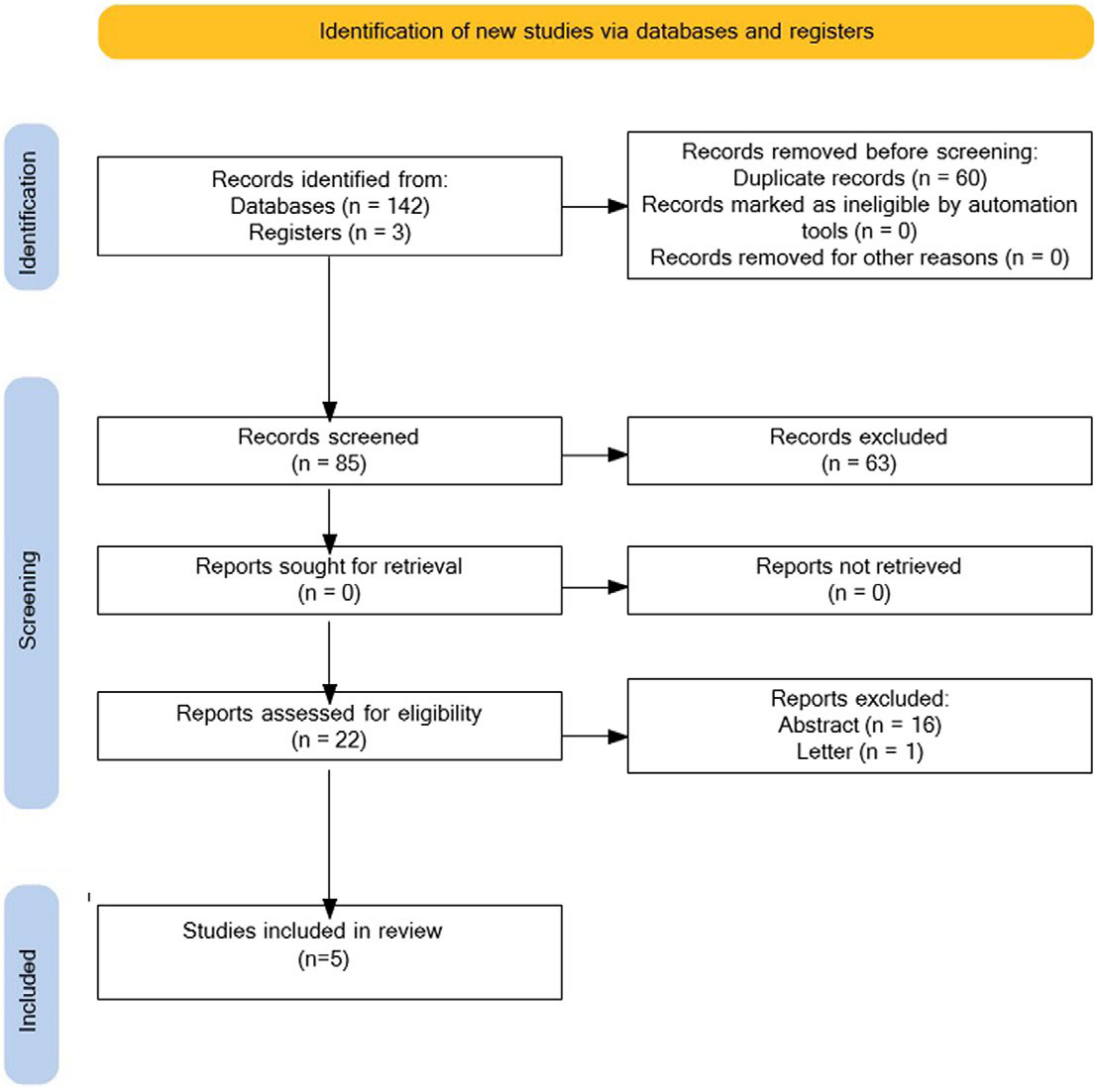
PRISMA flow diagram.

For the systematic review, 145 references were identified; after the exclusion of duplicates, 85 references were screened by title and abstract. Finally, five primary studies were selected. The main reason for exclusion was the format of publication. A detailed diagram of the selection processes is shown in Figure 1.

### Characteristics and Quality of the Studies

The five primary studies comprised two RCTs (HERCULES and TITAN) and three real-world studies. The TITAN and HERCULES trials were phase II and phase III studies published in 2016 and 2019, respectively (30;31). Both were double-blind, placebo-controlled, and multicenter, in which patients were randomized to receive caplacizumab or placebo in addition to daily plasma exchange and immunosuppression (Table 2).

**Table 2.** Characteristics of the included studies

Author	Year	Design	Country	Sample size		Age (Years) Median (range)		Females (%)		Use of rituximab *n* (%)		Treatment with caplacizumab (days)
				I^a^	C^b^	I^a^	C^b^	I^a^	C^b^	I^a^	C^b^	
Peyvandi et al. (30)	2016	Randomized clinical trial	Multicentric	36	39	41 (19–72)	42 (21–67)	24 (67)	20 (51)	2 (6)	9 (23)	38^c^
Scully et al. (31)	2019	Randomized clinical trial	Multicentric	72	73	45 (18–77)	47 (21–79)	49 (68)	51 (70)	28 (39)	35 (48)	35^d^
Völker et al. (32)	2020	Observational	Germany	60	–	45.7 (22–83)	–	42 (70)	–	48 (80)	–	34^d^
Coppo et al. (33)	2021	Observational	France	90	180	45^e^ (34–57)	43^e^ (30-57)	63 (70)	127 (70)	90 (100)	123 (68)	33^d^
Dutt et al. (34)	2021	Observational	United Kingdom	85	39	46 (3–82)	45 (15–93)	56 (66)	31 (80)	84 (99)	34 (87)	32^d^

a Intervention group: Caplacizumab + plasma exchange + glucocorticoids, with or without another immunosuppressant.

b Control group: Placebo + plasma exchange + glucocorticoids, with or without another immunosuppressant.

c Mean.

d Median.

e Median, (*Q1–Q3*).

The real-world studies were two retrospective observational studies and one prospective study (32–34). In these, information was collected and analyzed from medical records of patients who received caplacizumab plus standard treatment in countries such as Germany, France, and the United Kingdom, between 2018 and 2020. In addition, two of these studies compared the outcomes of patients who received the technology of interest with historical cohorts of patients treated with the standard regimen between 2014 and 2018, prior to the authorization of caplacizumab (33;34) (Table 2). Detailed information on the included studies is presented in Supplementary Tables 5 and 6. All studies evaluated at least one outcome of interest.

The quality rating of the HERCULES study obtained using the RoB2 tool was “some concerns.” However, three of the five domains of the tool were classified as “low risk of bias.” The TITAN study was rated as “high risk of bias” (Supplementary Table 7) (Supplementary Figure 1). The rating of the observational studies obtained using the tool from the Joanna Briggs Institute was low to moderate. The results of the qualification are described in detail in Supplementary Table 8.

Finally, due to the high risk of bias of one of the two RCTs included, a paired meta-analysis was not considered. Therefore, the main data presented in this document correspond to a narrative synthesis of the findings of the HERCULES clinical trial for each of the outcomes of interest and of the results of the real-world studies published to date.

### Results of Effectiveness, Efficacy, and Safety

In terms of effectiveness, the HERCULES trial showed a statistically significant reduction in the time to normalization of platelet count in patients receiving caplacizumab. In addition, these patients had a higher probability of achieving a response to treatment (Hazard ratio = 1.55), compared with those with the standard regimen (Table 3) (31).

**Table 3.** Results of effectiveness of included studies

HERCULES (31)			
Outcome	Caplacizumab^a^ (*n* = 72)	Standard regimen^b^ (*n* = 73)	*P*-value
Response time to treatment; median (95% CI) – Days	2.69 (1.89–2.83)	2.88 (2.68–3.56)	0.01
Composite outcome			
aTTP-related death, aTTP recurrence, or a major thromboembolic event during the treatment period of the trial	9 (12)	36 (49)	<0.001
aTTP-related death, *n* (%)	0	3 (4)	–
Recurrence during the treatment period (exacerbation), *n* (%)	3 (4)	28 (38)	–
Recurrence during the follow-up period (relapse), *n* (%)	6 (8)	0	–
Major thromboembolic event, *n* (%)	6 (8)	6 (8)	–
Refractoriness	0	3 (4)	0.06
General recurrence during the period of the study (exacerbation and relapse), *n* (%)	9 (12)	28 (38)	<0.001
Utilization of resources			
Number of days of PE			
Mean (95% CI)	5.8 (4.8–6.8)	9.4 (7.8–11.8)	–
Median (range)	5.0 (1.0–35.0)	7.0 (3.0–46.0)	–
Volume of PE – Liters			
Mean (95% CI)	21.3 (18.1–24.6)	35.9 (27.6–44.2)	–
Median (range)	18.1 (5.3–102.2)	26.9 (4.0–254.0)	–
Number of days of hospitalization			
Mean (95% CI)	9.9 (8.5–11.3)	14.4 (12.0–16.9)	–
Median (range)	9.0 (2.0–37.0)	12.0 (4.0–53.0)	–
Number of days in ICU			
Patients admitted to ICU, *n* (%)	28 (39)	27 (37)	–
Mean (95% CI)	3.4 (2.6–4.2)	9.7 (5.3–14.1)	–
Median (range)	3.0 (1.0–10)	5.0 (1.0–47.0)	–
TITAN (30)			
Outcome	Caplacizumab^a^ (*n* = 36)	Standard regimen^b^ (*n* = 39)	
Response time to treatment, event rate ratio (95% CI)	2.20 (1.28–3.78)		0.005
Patients without PE before randomization			
Response time, median (95% CI) – Days	3.0 (2.7–3.9)	4.9 (3.2–6.6)	–
Confirmed response, *n* (%)	29 (81)	24 (62)	–
Data censored at 30 days, *n* (%)	5 (14)	11 (28)	–
Patients with PE before randomization			
Response time, median (95% CI) – Days	2.4 (1.9–3.0)	4.3 (2.9–5.7)	–
Confirmed response, *n* (%)	2 (6)	4 (10)	–
Data censored at 30 days, *n* (%)	0	0	–
Secondary results			
Exacerbation, *n* (%)	3 (8)	11 (28)	–
Relapse, *n* (%)			
During the 1-month follow-up period	8 (22)	0	–
During the 12-month follow-up period	11 (31)	3 (8)	–
Full remission after initial daily PE, *n* (%)	29 (81)	18 (46)	–
Number of days of PE, mean (range)			
During the daily PE period	5.9 (3–15)	7.9 (2–35)	–
During the entire period of treatment with the drug under study	7.7 (3–21)	11.7 (2–43)	–
During the first 30 days of follow-up	10.2 (4–29)	11.7 (2–43)	–
Volume of plasma during the daily PE period, mean-Liters	19.9	28.3	–
Volume of plasma during the entire period of treatment with the drug under study, mean-Liters	25.8	41.8	–
Coppo et al. (33)			
Outcome	Caplacizumab cohort^a^ (*n* = 90)	Historic cohort^c^ (*n* = 180)	
Composite outcome; death or refractoriness	2 (2.2%)	22 (12.2%)	0.01
Death, *n* (%)	1 (1.1%)	12 (6.7%)	0.06
Refractoriness, *n* (%)	1 (1.1%)	16 (9%)	0.01
Exacerbations, *n* (%)	3 (3.4%)	70 (38.9%)	<0.01
Response time to treatment, median (*Q1–Q3*) – Days	5 (4–6)	12 (6–17)	<0.01
Number of daily PEs until remission, median (*Q1–Q3*)	5 (4–7)	10 (6–16)	<0.01
Volume of plasma, median (*Q1–Q3*) – Liters	24.2 (18.3–30.2)	44.4 (26.3–74.3)	<0.01
Length of hospitalization, median (*Q1–Q3*) – Days	13 (9–19)	22 (15–30)	0.01
Thromboembolic events, *n* (%)	11 (12%)	20 (11.1%)	0.79
Dutt et al. (34)			
Outcome	Caplacizumab cohort^a^ (*n* = 85)	Historic cohort^c^ (*n* = 39)	
Response time to treatment, median (IQR) – Days	4^d^ (3–8)	6 (4–10)	0.011
Number of PE, median (IQR) – Days	7 (5–14)	9 (8–16)	0.007
Number of days of hospitalization, median (IQR)	12 (8–24)	14 (9–17)	0.62
TTP-related deaths, *n* (%)	5 (6)^e^	0 (0)	0.32
Venous thromboembolism	4 (5)	2 (5)	>.99
Völker et al. (32)			
Time to platelet normalization after initiation of caplacizumab^a^, days (*n* = 64)			
Median (range)	3.0 (1–13)	–	–
Mean (95% CI)	3.78 (3.19–4.38)	–	–
Length of hospital stay (*n* = 59)			
Median (range)	18 (5–79)	–	–
Mean (95% CI)	21.6 (18–25.2)	–	–
Length of stay in the ICU (*n* = 54)			
Median (range)	4 (0–46)	–	–
Mean (95% CI)	5.8 (3.8–7.7)	–	–
Days of PE under treatment with caplacizumab			
Median (range)	4 (0–22)	–	–
Mean (95% CI)	5.3 (4.2–6.4)	–	–

aTTP, acquired thrombotic thrombocytopenic purpura; CI, confidence interval; ICU, intensive care unit; PE, plasma exchange; Q1, First quartile; Q3, third quartile; RIC, interquartile range.

a Caplacizumab + plasma exchange + glucocorticoids, with or without another immunosuppressant.

b Placebo + plasma exchange + glucocorticoids, with or without another immunosuppressant.

c Plasma exchange + glucocorticoids, with or without another immunosuppressant.

d Based on 81 patients, taking into account that four did not achieve normalization.

e In four of five deaths, caplacizumab was introduced >48 hr (delayed) after PE initiation (3–21 days).

Treatment with caplacizumab also resulted in a lower proportion of patients with a composite outcome event (aTTP-related death, aTTP recurrence, or a thromboembolic event) compared with the standard regimen group (12 vs. 49 percent, a reduction of 74 percent) (31).

In addition, the proportion of patients with aTTP recurrence during the whole period of study was 67 percent lower in the caplacizumab group compared with the placebo group (standard regimen). Moreover, no patients treated with caplacizumab were refractory to the intervention compared with three patients in the placebo group (Table 3) (31).

The HERCULES study also reported descriptively that treatment with caplacizumab reduced the number of sessions of plasma exchange, the volume of plasma, and the duration in the intensive care unit and hospitalization (Table 3) (31).

Real-world studies also observed a reduction in the time to normalization of platelet count and the number of plasma exchange sessions in patients treated with caplacizumab compared to those in historical cohorts treated with the standard regimen (Table 3) (33;34).

In the study by Coppo et al. (33), a lower incidence of the composite outcome of death or refractoriness was observed in patients in the caplacizumab cohort compared with those in the historical cohort. At the individual level, there was a lower proportion of refractoriness and death in patients in the caplacizumab cohort, with a statistically significant difference only for refractoriness. Likewise, the proportion of patients with an exacerbation, the number of plasma exchange sessions, and the volume of total plasma used were significantly lower in the caplacizumab cohort compared with those in the historical cohort (Table 3).

In the studies of Völker et al. (32) and Dutt et al. (34), no comparison with a historical cohort was made for the outcome of aTTP recurrence. However, the proportion of patients with this event in the caplacizumab cohort was comparable to that reported in patients in the HERCULES trial (31). In the study by Völker et al. (32), once patients started treatment with caplacizumab, the median time to treatment response was 3 days and a median of 4 additional days of plasma exchange was required – results that are also comparable with those of the intervention group in the HERCULES study (31).

In terms of safety, during the overall period of the HERCULES trial, the proportion of patients experiencing an adverse event was 96 percent in the caplacizumab group and 90 percent in the standard regimen group (Supplementary Table 9). The most common events in the caplacizumab group included fatigue, pyrexia, nausea, and gingival bleeding. The risk for the majority of adverse events was similar between the two groups: however (Supplementary Tables 9 and 10), the risk of bleeding and epistaxis was greater in patients receiving caplacizumab (31) (Supplementary Table 11). (Supplementary Tables 9, 10 and 11)

Bleeding events were reported in 65 percent of patients in the intervention group and in 48 percent of patients in the placebo group, with gingival bleeding and epistaxis being the most frequent in the caplacizumab group (Supplementary Table 11). All of these events were resolved, the majority without intervention. These events were mild or moderate in severity in the majority of patients and were classified as severe in three patients in the caplacizumab group and one patient in the placebo group. In eight patients in the caplacizumab group and in one patient in the placebo group, the bleeding event was classified as serious, epistaxis being the most frequent event among them. Only one patient received von Willebrand factor concentrate as the only treatment for resolution of the adverse event. Ultimately, five patients in the caplacizumab group and nine patients in the standard regimen group discontinued treatment due to an adverse event (31).

In the real-world studies, epistaxis and gingival bleeding were the most frequently observed bleeding events in patients treated with caplacizumab. These events were mostly resolved without a specific intervention (Supplementary Tables 12 and 13) (32–34).

The safety profile and the efficacy results of the HERCULES study are consistent with those reported in the TITAN clinical trial (30).

### Therapeutic Thresholds

The therapeutic thresholds for fourteen outcomes were determined through the deliberative process with the panel of experts (Table 4). The results of the voting and the guide questions for determination are presented in Supplementary guide questions (Supplementary Tables 14 and 15).

**Table 4.** Therapeutic thresholds

Outcome	Measure of association^a^ (95% IC)	Therapeutic threshold	Values obtained in the evidence (95% IC)	Reached
Effectiveness results^b^				
Response to treatment	RR: 1.55 (1.09–2.19)	8 patients more than comparator per 100 patients	48 more per 100 (from 8 more to 100 more)	Yes
Response time to treatment	DM = 2 days	Minimum reduction of 3 days in median response time to treatment	2-day reduction in median response time to treatment	No
Recurrences (exacerbation and relapse)	RR:0.33 (0.17–0.64)	25 fewer patients than comparator per 100 patients	26 fewer per 100 (from 14 fewer to 32 fewer)	No
Composite outcome (1) (aTTP-related death, aTTP recurrence, or at least one major thromboembolic event during the treatment period of the clinical trial)	RR:0.25 (0.13–0.49)	25 fewer patients than comparator per 100 patients	37 fewer per 100 (from 25 fewer to 43 fewer)	Yes
Composite outcome (2) (Death or refractoriness within 30 days of diagnosis)	RR: 0.18 (0.04–0.75)	7 fewer patients than comparator per 100 patients	10 fewer per 100 (from 3 fewer to 12 fewer)	No
Refractoriness	RR: 0.12 (0.02–0.93)	6 fewer patients than comparator per 100 patients	8 fewer per 100 (from 1 fewer to 9 fewer)	No
Exacerbation	RR: 0.09 (0.03–0.26)	24 fewer patients than comparator per 100 patients	36 fewer per 100 (from 29 fewer to 38 fewer)	Yes
Number of days of plasma exchange	DM = 5 days fewer	Minimum reduction of 5 days in median hospital stay	5-day reduction in the median number of days of plasma exchange	Yes
Number of days of hospitalization	DM = 9 days fewer	Minimum reduction of 8 days in median hospital stay	9-day reduction in median hospital stay	Yes
Safety results^c^				
Serious adverse events	1.98 (1.06–3.7)	15 patients more than comparator per 100 patients	16 more per 100 (from 1 more to 44 more)	No
Bleeding adverse events	1.35 (1.01–1.81)	20 patients more than comparator per 100 patients	17 more per 100 (from 0 fewer to 39 more)	No
Serious bleeding adverse events	8.23 (1.06–64)	1 patient more than comparator per 100 patients	10 more per 100 (from 0 fewer to 86 more)	No
Gingival bleeding	13.4 (1.8–99.5)	20 patients more than comparator per 100 patients	17 more per 100 (from 1 more to 100 more)	No
Epistaxis	11.9 (2.9–48)	18 patients more than comparator per 100 patients	30 more per 100 (from 5 more to 100 more)	No

aTTP, acquired thrombotic thrombocytopenic purpura; CI, confidence interval; DM, difference of medians; RR, relative risk.

a In accordance with the IETS manual, the outcomes with a statistically significant difference were submitted.

b For dichotomous (yes/no) and continuous effectiveness outcomes in which the threshold was not met (“no” in Reached column), it is understood that caplacizumab together with the standard regimen offers the same clinical benefits as its comparator (standard regimen). That is, it is considered neither superior nor inferior at the clinical level.

c For safety outcomes for which the threshold was not met (“no” in Reached column), caplacizumab plus the standard regimen is understood to be not inferior in terms of safety than its comparator (standard regimen). That is, at the clinical level they present a similar safety profile.

Using the established thresholds and the effectiveness results of the technology of interest, the clinical superiority of treatment with caplacizumab compared to the standard regimen was determined for the outcomes of decreased incidence of exacerbations and the composite outcome of aTTP-related death, aTTP recurrence or at least one major thromboembolic event. Likewise, it was determined that the treatment with caplacizumab presents an additional therapeutic benefit in achieving the normalization of the platelet count, and the reduction of the number of days of plasma exchange and the hospital stay, in comparison with the standard regimen. For the other effectiveness outcomes, caplacizumab reached or exceeded the threshold point in most cases: however, the lower CI did not allow for the conclusion of superiority over the standard regimen, as it was not equal to or higher than the threshold (Table 4).

In terms of safety, the results reported for caplacizumab did not exceed any of the established thresholds, indicating that the expected number of patients was not reached in order to conclude that treatment with caplacizumab plus the standard regimen is less safe than the standard regimen (Table 4).

### Acceptability of the Effect of the Technology

In consensus, the panel established that the effect of the technology in all the evaluated outcomes is acceptable, stating that they were willing to use the technology of interest, considering the balance of benefits and risks. The results of the voting are detailed in Supplementary Tables 16 and 17.

### Classification of the Technology

Based on the information presented in the input matrix, the panel determined through consensus that caplacizumab offers a better benefit–risk balance than its comparator for treatment response and the composite outcome, that is, it is *superior* to the standard regimen, with a *low* certainty of evidence, according to the GRADE methodology (Supplementary Table 18).

For recurrences, serious adverse events, bleeding events, epistaxis, and gingival bleeding, the panel reached a consensus that caplacizumab offers a benefit–risk balance similar to that of the comparator, that is, it is *no different* from the standard regimen, with a *low* certainty of evidence.

For the outcomes of refractoriness, exacerbations, composite outcome (death or refractoriness), response time to treatment, and the number of days of plasma exchange and of hospitalization, the certainty of the evidence was *very low*, this being the causal factor for non-pronouncement by part of the panel, so these outcomes were not classified.

Ultimately, the panel determined that overall treatment with caplacizumab in combination with the standard regimen is *superior* to the standard regimen for the treatment of patients with aTTP, with a *low* certainty of evidence. The results of the voting are detailed in Supplementary Table 19.

## Discussion

In the present evaluation through a systematic and exhaustive search of the literature, two RCTs (TITAN and HERCULES) and three real-world studies were identified which evaluated the administration of caplacizumab in patients with aTTP (30–34).

The results of the HERCULES study showed that treatment with caplacizumab reduces the time to normalization of platelets. Also, the addition of caplacizumab to the standard therapy resulted in a lower proportion of patients with recurrence and the composite outcome of death, refractoriness, or a major thromboembolic event as well than with standard therapy. These results were consistent with those reported in the TITAN study (31).

A possible bias in the HERCULES clinical trial is the imbalance in the baseline characteristics of the patients regarding the use of rituximab and some factors that showed a more severe condition in the patients in the caplacizumab group. However, different stratified analyzes of the data have shown that treatment with caplacizumab improved clinical outcomes, regardless of the type of initial immunosuppression and the severity of aTTP (35;36).

Moreover, the findings from the real-world studies confirm the results of the HERCULES clinical trial and are consistent with them (7;13;32–34;37). In the study of Völker et al. (32), the median time to normalization was very similar to that reported in the HERCULES trial (29). Likewise, in the studies of Coppo et al. (33) and Dutt et al. (34), it was shown that patients treated with caplacizumab achieved normalization of platelet count in a shorter time than their historical controls. Coppo et al. (33) also observed a lower proportion of patients with a composite outcome (death or refractoriness) or with an exacerbation in the caplacizumab cohort. These results have also been replicated in a real-life study carried out in Spain by Izquierdo et al. (38), which has been recently published. In this study, the data of the patients who were treated with caplacizumab together with the standard regimen were compared with a cohort of patients who received the standard treatment prior to the introduction of this molecule, finding a lower incidence of exacerbations, refractoriness, and death, the first two with statistically significant differences, in patients treated with caplacizumab.

Izquierdo et al. (38), also found that among patients who received caplacizumab as an initial treatment, the clinical response time was significantly shorter in patients treated within the first 3 days compared to those who started later.

Coppo et al. (33), Dutt et al. (34), and Izquierdo et al. (38) used a statistical test for the comparison of outcomes related to the use of medical care resources, where it was shown that treatment with caplacizumab significantly reduced the number of plasma exchange sessions, the volume of plasma, and the hospital stay.

In the real-world studies, a higher proportion of patients received concomitant caplacizumab and rituximab, compared to patients in the HERCULES trial. However, evidence shows that rituximab enhances and stabilizes long-lasting ADAMTS13 activity, generally after a period of 2–5 weeks (33). That is, rituximab becomes effective after an average time of 2 weeks once the first infusion is performed, and therefore the improvement observed in the early or acute stage of the disease could be attributable to the use of caplacizumab (34;37;38).

In terms of safety, the mechanism of action of caplacizumab exposes patients to an increased risk of mucocutaneous hemorrhage: however, most of these events are minor and are resolved without any intervention (39).

In addition, based on the deliberative processes and the findings of the scientific evidence, the clinical superiority of treatment with caplacizumab over the standard regimen was determined, in critical outcomes and therefore of high interest for clinical practice. Moreover, caplacizumab was not considered inferior in terms of safety compared to the standard regimen.

Based on the input matrix, the experts determined the superiority of treatment with caplacizumab, since it offers a better benefit–risk balance than the standard regimen for the treatment of patients with aTTP. Within the available inputs for the classification, the panel gave more value to the statistical estimates and the absolute effects of the technology according to the GRADE approach. This was due to the fact that the members of the panel considered that the process for determining the therapeutic threshold and the decision of clinical superiority is prone to subjectivity, and so they preferred to base their views on more objective data to determine a category.

The safety and efficacy of treatment with caplacizumab have also been demonstrated in long-term outcomes. The post-HERCULES study was recently published (40), in which the patients of the HERCULES study were followed for three more years. The results showed that the safety profile was similar to that previously reported and was equally effective for the control of recurrent episodes of aTTP. This study also included quality-of-life variables and showed that cognitive function and quality of life remained stable or improved during the 3-year follow-up in patients who had also received caplacizumab. No important cases of organ dysfunction were observed either, since the markers were found within the normal range in almost all the patients. According to the authors, these data demonstrate that in the long term, caplacizumab, including its repeated administration for new episodes, is safe and effective for the treatment of aTTP episodes.

Currently, due to the results found in the scientific evidence, caplacizumab has approval for the treatment of the condition of interest by the EMA and the FDA (20;21) and it is included as part of the first-line treatment, in the latest international guidelines published on the management of aTTP (19). Additionally, results from real-world studies have led to caplacizumab being incorporated as part of the standard treatment of patients with aTTP in several European countries (13), including France (33;37), Germany (32), the United Kingdom (34), and Spain (7).

## Conclusion

The data presented show that the addition of caplacizumab to the standard regimen reduces the time to the normalization of platelet count, recurrence, and refractoriness, substantially reducing the utilization of healthcare resources. Although the additional benefit of caplacizumab is associated with an increased incidence of mild and moderate mucocutaneous bleeding, these are considered self-limiting. In light of this evidence and the established therapeutic value thresholds, the overall perception of the experts consulted is that caplacizumab offers a better benefit–risk balance, is superior in some critical outcomes of effectiveness, and is no different in outcomes of safety, and therefore can be considered to be superior overall to the standard regimen for the treatment of patients with acute episodes of aTTP.

In the Latin American region, decisions related to the availability, price, and reimbursement of health technologies are increasingly being based on HTA. This is in order to make healthcare decisions based on the best available scientific evidence, which can guide the use of the health system’s finite resources. Based on the experience with the evaluation and classification of caplacizumab following the Colombian methodology, it is suggested to include in the evaluation methodologies of the region, deliberative processes for the determination of the threshold of therapeutic value, acceptability, and classification of the technology, with which the value (effectiveness and safety) of the technologies of interest can be determined considering other aspects beyond statistical significance.

## Supplementary material

The supplementary material for this article can be found at https://doi.org/10.1017/S0266462323000442.
